# Commentary: Rheumatoid arthritis and the risk of end-stage renal disease: a nationwide, population-based study

**DOI:** 10.3389/fmed.2023.1194649

**Published:** 2023-05-25

**Authors:** Qingfeng Peng, Gang Wang, Jie Li

**Affiliations:** ^1^Department of Nephrology, Zhuzhou Hospital Affiliated to Xiangya Medical College, Central South University, Zhuzhou, Hunan, China; ^2^Department of Rheumatology and Immunology, Zhuzhou Hospital Affiliated to Xiangya Medical College, Central South University, Zhuzhou, Hunan, China

**Keywords:** rheumatoid arthritis, end-stage renal disease, chronic kidney disease, nationwide population-based study, risk factor

We read with interest the recent publication by Suh et al. on the rheumatoid arthritis (RA) and the risk of end-stage renal disease (ESRD): a nationwide, Population-based study (1). The authors conclude that RA increase the risk of ESRD. As the risk of ESRD imposed by RA is relatively higher in relatively young and healthy individuals, kidney-protective treatment, such as biologic agents, should be preferentially considered among these patients with RA (1). We support and appreciate the authors' work and agree with their conclusions, but have some concerns about some of the details in the article.

Firstly, in recent years, the pathogenic role of inflammation in RA-related kidney damage has been increasingly studied. Two prospective studies of patients with inflammatory arthritis have shown that the severity of arthritis and the duration of inflammation are important risk factors for chronic kidney disease (CKD) (2, 3). A 5-year follow-up study by Chiu et al. demonstrated that even after adjusting for traditional cardiovascular risk factors, RA patients still have a higher risk of developing CKD compared to those without RA, and chronic inflammation is considered one of the main factors contributing to the increased risk of CKD in RA patients (4). A retrospective study by Kochi et al. showed that persistently high levels of CRP, even after adjusting for classic CKD risk factors, remain an important risk factor for CKD in RA patients (5). A Japanese cohort study found a close association between elevated CRP levels and the incidence of CKD, with sustained elevation of CRP for at least 6 months being an important risk factor for CKD in RA patients (6). In summary, disease activity and related inflammatory status of RA are important confounding factors in this study, and we recommend that patients be evaluated for indicators of RA disease activity such as CRP, ESR, CDAI, SDAI, and DAS28-ESR.

Secondly, RA therapeutic drugs are generally believed to cause renal damage, including non-steroidal anti-inflammatory drugs (NSAIDs), nephrotoxic disease-modifying antirheumatic drugs (DMARDs), and corticosteroids. A cohort study by Chiu et al. including 12,579 RA patients from the Taiwan National Health Insurance database showed a close correlation between NSAID use and increased risk of CKD in RA patients (4). A retrospective cohort study by Tokoroyama showed that in 813 patients without prior CKD, the cumulative incidence of CKD increased to 59.5% over time, and in addition to well-known cardiovascular risk factors, the use of prednisolone and NSAIDs increased the risk of death (7). Among DMARDs, gold, penicillamine, or bucillamine may be associated with proteinuria formation (8, 9). Calcineurin inhibitors such as cyclosporine and tacrolimus can induce a decrease in glomerular filtration rate (GFR) (10). There are also reports describing TNF-α inhibitors not only effective in treating active RA but also stabilizing or improving kidney function by reducing secondary amyloidosis (11). Therefore, drugs are an important confounding factor in this study and should be further clarified.

Thirdly, research has shown that renal impairment in RA is associated with the presence of elevated serum uric acid levels and extra-articular diseases (12). The potential link between uric acid and renal impairment may be mediated by hyperuricemia-related cardiovascular diseases, hypertension, and insulin resistance (13). Similarly, the presence of extra-articular diseases in RA, such as interstitial pulmonary fibrosis and systemic vasculitis, has been found to be an independent predictor of renal impairment (12). This is explained by the fact that RA patients with extra-articular manifestations may have higher disease activity and are more likely to develop amyloidosis of the kidneys. Therefore, this study should clarify the impact of RA-related comorbidities such as interstitial pulmonary fibrosis and systemic vasculitis on the study.

Finally, GFR is the best indicator for assessing kidney function, and many equations have been used in recent decades to estimate GFR. In this study, the authors chose the MDRD (Modification of Diet in Renal Disease) equation instead of the CKD-EPI (Chronic Kidney Disease Epidemiology Collaboration) equation. Compared to the CKD-EPI equation, the MDRD study equation may overestimate the incidence of CKD, while the CKD-EPI equation performs better in terms of bias, study performance, and accuracy (14). Meanwhile, muscle wasting is a common feature of RA patients, so serum creatinine levels will inevitably be influenced by the muscle mass of RA patients, and eGFR values calculated using serum creatinine levels may underestimate the actual kidney function of RA patients.

In conclusion, before these issues are clarified, this study's findings should be interpreted cautiously.

## Author contributions

QP and GW wrote the paper. JL reviewed and edited the manuscript. All authors had access to the data. All authors contributed to the article and approved the submitted version.
